# Expression of Concern: Epistatic interactions between *PHOTOPERIOD1*, *CONSTANS1* and *CONSTANS2* modulate the photoperiodic response in wheat

**DOI:** 10.1371/journal.pgen.1011097

**Published:** 2023-12-20

**Authors:** The PLOS Genetics Editors

Following the publication of this article [1], concerns were raised regarding results presented in the originally published Fig 5, provided with this notice in S2 File. Specifically, the corresponding author informed PLOS that the underlying qRT-PCR expression data reported in this study were inappropriately manipulated.

The University of California, Davis confirmed that author DPW admitted to manipulation of the data underlying the results presented in the originally published Fig 5 (see S2 File).

The authors replicated the qRT-PCR experiments presented in Fig 5 by regrowing the same plant materials in growth chambers under the same long-day (LD) and short-day (SD) photoperiods and temperature conditions. The RNA was extracted at the same developmental stage as the original study, and the expression profiles of the genes were re-analysed by quantitative RT-PCR using the same primers. The original raw data for the repeat experiments are in S1 File of this notice.

The authors state that the major conclusions reported in the original study remain unaffected, but that the replicate experiments highlighted some differences in the results reported in the originally published Fig 5 (see S2 File) and corresponding Results sections. The specific changes to the article’s results are described in Table 1 below.

**Table 1:** 

Figure	Revised result (based on repeat experiment data)	Original Results
5A	In Kronos-PS, *CO1* showed a peak at ZT12 under LD (in the light phase) and SD (in the dark phase).	In the same genotype, the *CO1* peak under SD was at ZT16.
5C, LD	*PPD1* showed a peak at ZT12 in Kronos-PS and was downregulated in *co1 co2* at ZT8 and ZT12.	PPD1 peaked at ZT8 and ZT16 in Kronos-PS and was not downregulated in *co1 co2*.
5C, SD	*PPD1* showed a peak at ZT4 in Kronos-PI.	*PPD1* showed a peak at ZT8 in Kronos-PI
5DE, LD	Better correlation between *FT1* and *VRN1* transcription profiles.	Limited correlation between *FT1* and *VRN1* transcription profiles.
5E, SD	*VRN1* transcripts were significantly higher in *ppd1* relative to other genotypes at ZT0 and ZT8.	*VRN1* transcripts were significantly higher in *ppd1* only at ZT0.
5F, LD	*VRN2* was significantly downregulated in *ppd1*, *co1* and *co2* at the ZT12 peak relative to Kronos-PS.	No downregulation of *VRN2* in *ppd1*, *co1* and *co2* relative to Kronos-PS at ZT12.

In light of these differences, at the time of publication of this Expression of Concern, this article [1] was republished to update Fig 5 and multiple paragraphs of the Results subsection with the heading Effect of mutations in PPD1, CO1 and CO2 on the transcriptional profiles of flowering genes, and the Discussion subsection with the heading Complex interactions at the transcriptional level contribute to the observed genetic interactions on heading time. Please download this article again to view the correct version. The original version of the published article remains available in S2 File provided with this notice. S3 File shows the changes made to the article’s text at the time of republication.

The PLOS Publication Ethics team reviewed this case in collaboration with the *PLOS Genetics* Editors, and carefully considered case details including the confirmed data manipulation, that the data in question comprise a relatively minor portion of the results, and the reliability of the article’s main findings as demonstrated by the repeat data and supported by an Editorial Board member. PLOS issues this Expression of Concern to inform readers of the data manipulation concerns and that findings in the originally published Fig 5 (see S2 File) in [1] are unreliable. However, the journal stands by the article once amended with this notice to include these alerts and the data obtained in repeat experiments.

The sequence data have been deposited in GenBank with accession numbers MT043302 (CO-A1), MT043303 (CO-B1), MT043304 (CO-A2), and MT043305 (CO-B2).

Mutant lines are available for distribution from multiple copies of the TILLING populations distributed worldwide, with specific locations in the John Innes Centre and the University of California Davis.

The supporting data for the modified Fig 5 is provided in S1 File available with this notice.

## Supporting information

S1 File Underlying data for the replicate experiments presented in the updated Fig 5. (XLSX)

S2 File Originally published PDF of this article [1]. (PDF)

S3 File Manuscript with tracked changes to highlight updates to results and discussion section following repeat experiments. (DOCX)
