# Supplementary material for: Epistatic interactions between PHOTOPERIOD1, CONSTANS1 and CONSTANS2 modulate the photoperiodic response in wheat
Source: PLoS Genet. 2020 Jul 13;16(7):e1008812. doi: 10.1371/journal.pgen.1008812 (PMC7394450; doi:10.1371/journal.pgen.1008812)
Supplement: S2 Fig — (A) Kronos-ppd1 null mutant and (B) Kronos-PS control plants grown under SD and dissected 140 days (20 weeks) after sowing. Note the faster development of Kronos-PS relative to Kronos-ppd1-null. In both genotypes spikes failed to emerge before 180 days when the experiment was terminated. (PDF) [file pgen.1008812.s002.pdf]

S2 Fig. Dissection of developing spikes. (**A**) Kronos-*ppd1* null mutant and (**B**) Kronos-PS control plants grown under SD and dissected 140 days (20 weeks) after sowing. Note the faster development of Kronos-PS relative to Kronos-*ppd1*-null. In both genotypes spikes failed to emerge before 180 days when the experiment was terminated.

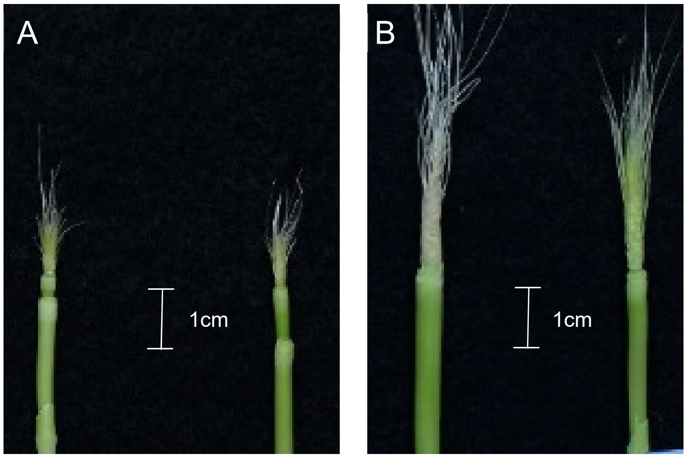

Kronos-*ppd1*-null Kronos-PS
