# Supplementary material for: Epistatic interactions between PHOTOPERIOD1, CONSTANS1 and CONSTANS2 modulate the photoperiodic response in wheat
Source: PLoS Genet. 2020 Jul 13;16(7):e1008812. doi: 10.1371/journal.pgen.1008812 (PMC7394450; doi:10.1371/journal.pgen.1008812)

S3 Fig. Effect of *ppd1*, *co1* and *co2* loss-of-function mutations and photoperiod on *CO1* and *CO2* transcript levels. RNA samples were collected at ZT4 from leaves of six-week-old Kronos-PS and *ppd1* plants (with and without *co1* and *co2*). (**A**) *CO1* transcript levels. (**B**) *CO2* transcript levels. Dunnett's tests were used to compare the two mutants with the wild type. Transcript levels are expressed relative to *ACTIN* using the ΔCt method. Averages and standard errors of the means were calculated from a minimum of five biological replicates per genotype.

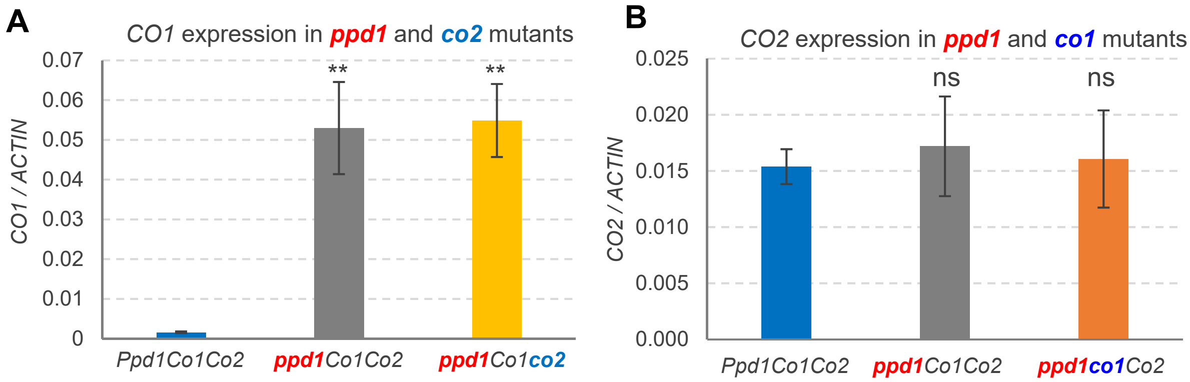

Supplement: S3 Fig — RNA samples were collected at ZT4 from leaves of six-week-old Kronos-PS and ppd1 plants (with and without co1 and co2). (A) CO1 transcript levels. (B) CO2 transcript levels. Dunnett’s tests were used to compare the two mutants with the wild type. Transcript levels are expressed relative to ACTIN using the ΔCt method. Averages and standard errors of the means were calculated from a minimum of five biological replicates per genotype. (PDF) [file pgen.1008812.s003.pdf]
