# Supplementary material for: Epistatic interactions between PHOTOPERIOD1, CONSTANS1 and CONSTANS2 modulate the photoperiodic response in wheat
Source: PLoS Genet. 2020 Jul 13;16(7):e1008812. doi: 10.1371/journal.pgen.1008812 (PMC7394450; doi:10.1371/journal.pgen.1008812)
Supplement: S4 Fig — Results extracted from a published RNAseq study [32]. Samples were collected from the newest expanded leaves at ZT4 from 4w-old plants under LD (n = 4) and 8w-old plants under SD (n = 8) to synchronize wild type genotypes grown under different photoperiods to a similar early reproductive stage (W3 early spike development without terminal spikelet). Comparisons between LD and SD should be interpreted with caution, since photoperiod effects and chronological time effects are conflated. Factorial ANOVAS were performed separately for each gene and P values for photoperiod (SD vs LD), genotype (WT, phyB, phyC) and their interactions are indicated below the gene names. (PDF) [file pgen.1008812.s004.pdf]

S4 Fig. Effect of *phyB*-null and *phyC*-null mutations in Kronos-PI on the transcriptional profiles of *CO1* and *CO2* under SD and LD. Results extracted from a published RNAseq study [32]. Samples were collected from the newest expanded leaves at ZT4 from 4w-old plants under LD (n = 4) and 8w-old plants under SD (n = 8) to synchronize wild type genotypes grown under different photoperiods to a similar early reproductive stage (W3 early spike development without terminal spikelet). Comparisons between LD and SD should be interpreted with caution, since photoperiod effects and chronological time effects are conflated. Factorial ANOVAS were performed separately for each gene and *P* values for photoperiod (SD vs LD), genotype (WT, *phyB*, *phyC*) and their interactions are indicated below the gene names.

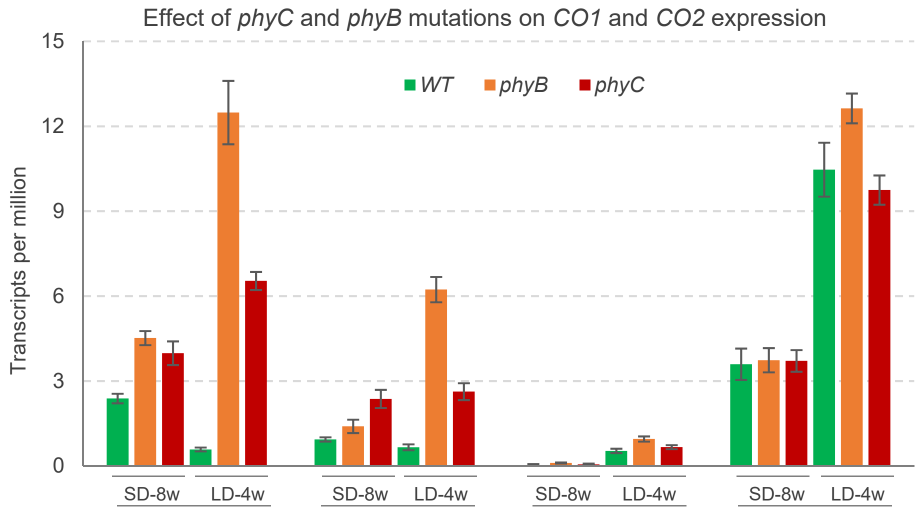

	CO-A1	CO-B1	CO-A2	CO-B2
Photoperiod	0.0003	<0.0001	<0.0001	<0.0001
Genotype	<0.0001	<0.0001	ns	ns
Interaction	<0.0001	<0.0001	ns	ns
