# Supplementary material for: Epistatic interactions between PHOTOPERIOD1, CONSTANS1 and CONSTANS2 modulate the photoperiodic response in wheat
Source: PLoS Genet. 2020 Jul 13;16(7):e1008812. doi: 10.1371/journal.pgen.1008812 (PMC7394450; doi:10.1371/journal.pgen.1008812)

S5 Fig. Yeast-two-hybrid (Y2H) assays. Primers for cloning *PPD1* (from Kronos) and PHYB truncations (from *T. monococcum*) are listed in Supplementary Table S5. Primers and vectors for *CO1*, *CO2* and *VRN2* are described in [33] and those for *PHYC* and full-length *PHYB* in [15]. Transformants were selected on SD medium lacking leucine (L) and tryptophan (W) plates and re-plated on SD medium lacking L, W, histidine (H) and adenine (A) to test the interactions. Due to auto-activation, CO1 and CO2 can only be used as preys. Only N-PHYB can be used as bait without autoactivation, so this is the only PHYB clone tested for interactions with CO1 and CO2. For VRN2, we used the functional ZCCT1 paralog from *T. monococcum*. The PPD1-bait construct used in assays presented in this figure is the same as in Figure 7A showing a positive interaction with both CO1 and CO2.

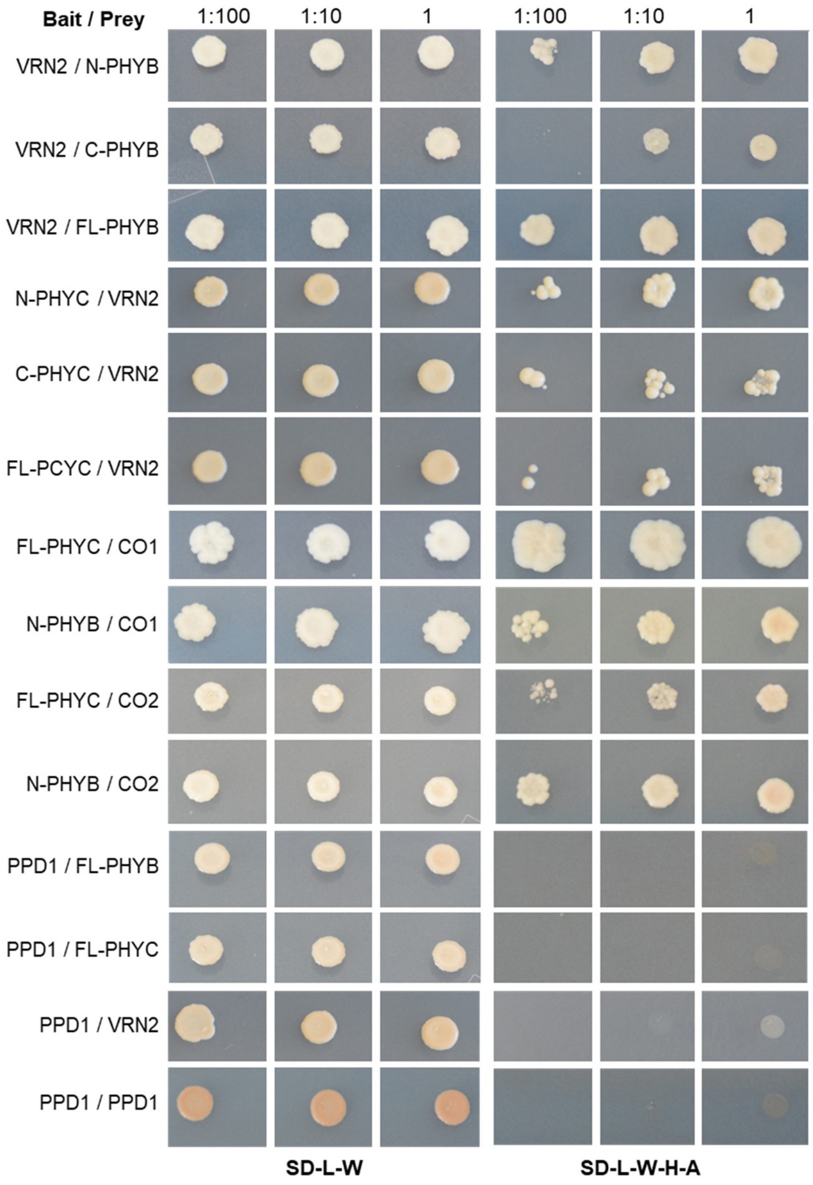

Supplement: S5 Fig — Primers for cloning PPD1 (from Kronos) and PHYB truncations (from T. monococcum) are listed in Supplementary Table S5. Primers and vectors for CO1, CO2 and VRN2 are described in [33] and those for PHYC and full-length PHYB in [15]. Transformants were selected on SD medium lacking leucine (L) and tryptophan (W) plates and re-plated on SD medium lacking L, W, histidine (H) and adenine (A) to test the interactions. Due to auto-activation, CO1 and CO2 can only be used as preys. Only N-PHYB can be used as bait without autoactivation, so this is the only PHYB clone tested for interactions with CO1 and CO2. For VRN2, we used the functional ZCCT1 paralog from T. monococcum. The PPD1-bait construct used in assays presented in this figure is the same as in Fig 7A showing a positive interaction with both CO1 and CO2. (PDF) [file pgen.1008812.s005.pdf]
