# Supplementary material for: Epistatic interactions between PHOTOPERIOD1, CONSTANS1 and CONSTANS2 modulate the photoperiodic response in wheat
Source: PLoS Genet. 2020 Jul 13;16(7):e1008812. doi: 10.1371/journal.pgen.1008812 (PMC7394450; doi:10.1371/journal.pgen.1008812)
Supplement: S1 Table — We sequenced genome-specific PCR products the first time to confirm amplification of the correct target. (PDF) [file pgen.1008812.s006.pdf]

S1 Table. Genome-specific primer sequences and PCR conditions for TILLING. We sequenced genome-specific PCR products the first time to confirm amplification of the correct target.

Gene name	Target	Primer name	Primer sequence (5' to 3')	Product (bp)	Ann. Temp (ºC)	Extension time	Enzyme
CO1A	Exon1	CO1A-5P-F1	ACATAGGCAGTGCATGAACACAT	1174	59*	1 m 30 s	*Hpy*188I
		CO1A-5P-R2	AGAAGTAGAAAAAGTTGAAGAAAGAG				
CO1A	Exon 2	CO1A-3P-F2	CAATTCATCTCTAGGAAAGTAC	955	54*	1 m 30 s	-
		CO1A-3P-R1	CGTGCTATCTGAAACTATAAAC				
CO1B	Exon 1	CO1-5P-CF2	CCACTGACACCCTACTATTAG	1375	55*	1 m 30 s	-
		CO1B-5P-R2	AGAAGTGGAAAAAGTTGAAGAAAGAA				
CO1B	Exon 2	CO1B-3P-F2	CAATTCATCTCTAGGAAAGTAA	987	54*	1 m 30 s	*EcoR*V
		CO1B-3P-R1	CGTGCTATCTGAAACTATAAAT				
CO2A	Exon 1	CO2A-BBOX-F1	TTCCAACACTGACTGCTTCTG	1081	53*	1 m 30 s	-
		CO2-BBOX-CR1	CTTGTATCCTAAGTGAGATGTGAC				
CO2A	Exon 2	CO2A-ZCCT-F1	GGTTACAACTCTGGATGGTAG	922	55	1 m 30 s	*EcoR*V
		CO2-ZCCT-CR4	GGACTATGTGGTTCACAATATG				
CO2B	Exon 1	CO2B-BBOX-F1	TTCCAACACTGACTGCTCCCA	1021	54*	1 m 30 s	-
		CO2-BBOX-CR1	CTTGTATCCTAAGTGAGATGTGAC				
CO2B	Exon 2	CO2B-ZCCT-F1	CTGTCCAACAGAATGTTTGAC	931	54*	1 m 30 s	-
		CO2-ZCCT-CR3	CTAACAGTAGAAGTCCCAACC				

*Touch-down protocol for PCR includes an additional 94 ºC for 5 m, 12 cycles of initial touch-down, with a reduction of 0.5 ºC per cycle (6 ºC total from final annealing T), then followed by 40 cycles of 94 ºC for 5 m, annealing temperature for 30 s and a final extension time of 7 m at 72 ºC.
