# Supplementary material for: Epistatic interactions between PHOTOPERIOD1, CONSTANS1 and CONSTANS2 modulate the photoperiodic response in wheat
Source: PLoS Genet. 2020 Jul 13;16(7):e1008812. doi: 10.1371/journal.pgen.1008812 (PMC7394450; doi:10.1371/journal.pgen.1008812)
Supplement: S2 Table — (PDF) [file pgen.1008812.s007.pdf]

S2 Table. Number of mutations detected in the targeted regions of wheat *CO1* and *CO2* homologs in the Kronos TILLING population.

Gene	Mutations (Total)	Non-synonymous	Synonymous	Splice or stop mutations
CO-A1	37	14	22	1
CO-B1	49	28	20	1
CO-A2	23	9	13	1
CO-B2	44	23	19	2
Total	153	74	74	5
