# Supplementary material for: Epistatic interactions between PHOTOPERIOD1, CONSTANS1 and CONSTANS2 modulate the photoperiodic response in wheat
Source: PLoS Genet. 2020 Jul 13;16(7):e1008812. doi: 10.1371/journal.pgen.1008812 (PMC7394450; doi:10.1371/journal.pgen.1008812)
Supplement: S3 Table — This factorial ANOVA combined all four classes of CO1 and CO2 wild type and mutant alleles in photoperiod sensitive (PS, Ppd-A1b, three experiments) and photoperiod insensitive backgrounds (PI, Ppd-A1a, two experiments). For the statistical analyses, we used experiments as blocks nested within PPD1 classes. (PDF) [file pgen.1008812.s008.pdf]

S3 Table. Analysis of variance for heading time under long days (LD, 16 h light / 8 h darkness). This factorial ANOVA combined all four classes of *CO1* and *CO2* wild type and mutant alleles in photoperiod sensitive (PS, *Ppd-A1b*, three experiments) and photoperiod insensitive backgrounds (PI, *Ppd-A1a*, two experiments). For the statistical analyses, we used experiments as blocks nested within *PPD1* classes.

Dependent Variable: Heading time

Source	DF	Sum of Squares	Mean Square	*F* Value	*P* > F
Model	10	948.953	94.895	38.04	<.0001
Error	51	376.658	2.494		
Corrected Total	61	1325.611			

R^2=0.716

S3.A. Overall 3-way ANOVA.

Source	DF	Type III SS	Mean Square	*F* Value	*P* > F
Experiment	3	55.847	18.616	7.46	0.0001
PPD1	1	408.755	408.755	163.87	<.0001
CO1	1	173.578	173.578	69.59	<.0001
CO2	1	33.081	33.081	13.26	0.0004
*CO1*CO2*	1	2.250	2.250	0.90	0.3437
*PPD1*CO1*	1	55.066	55.066	22.08	<.0001
*PPD1*CO2*	1	78.353	78.353	31.41	<.0001
*PPD1*CO1*CO2*	1	20.670	20.670	8.29	0.0046

S3.B. 2-way ANOVA *CO1* x *CO2* in PS (Main text, Fig. 2B).

Source	DF	Type III SS	Mean Square	*F* Value	*P* > F
Exp	2	55.073	27.536	10.55	<.0001
CO1	1	249.762	249.762	95.67	<.0001
CO2	1	5.653	5.653	2.17	0.1448
CO1*CO2	1	21.494	21.494	8.23	0.0052

S3.C. 2-way ANOVA *CO1* x *CO2* in PI (Main text, Fig. 2C).

Source	DF	Type III SS	Mean Square	*F* Value	*P* > F
Exp	1	0.775	0.775	0.33	0.5668
CO1	1	14.386	14.386	6.16	0.0157
CO2	1	92.724	92.724	39.69	<.0001
CO1*CO2	1	4.036	4.036	1.73	0.1934
