# Supplementary material for: Epistatic interactions between PHOTOPERIOD1, CONSTANS1 and CONSTANS2 modulate the photoperiodic response in wheat
Source: PLoS Genet. 2020 Jul 13;16(7):e1008812. doi: 10.1371/journal.pgen.1008812 (PMC7394450; doi:10.1371/journal.pgen.1008812)
Supplement: S4 Table — All four allelic combinations for CO1 and CO2 wild type and mutant alleles were analyzed in a photoperiod insensitive background (Kronos-PI). (PDF) [file pgen.1008812.s009.pdf]

S4 Table. Analysis of variance for heading time under short days (SD, 8 h light / 16 h darkness). All four allelic combinations for *CO1* and *CO2* wild type and mutant alleles were analyzed in a photoperiod insensitive background (Kronos-PI).

Dependent Variable: Heading time

Source	DF	Type III SS	Mean Square	*F* Value	*P* > F
Model	3	1099.363	366.454	28.61	<.0001
Error	35	448.226	12.806		
Corrected Total	38	1547.590			

R^2 0.710

Source	DF	Type III SS	Mean Square	*F* Value	*P* > F
Main effects					
CO1	1	324.801	324.801	25.36	<.0001
CO2	1	574.507	574.507	44.86	<.0001
CO1 x CO2	1	131.177	131.177	10.24	0.0029
Simple effects					
CO1-co1 in *CO2* WT	1	20.056	20.056	1.57	0.2191
CO1-co1 in *co2* mut	1	470.028	470.028	36.70	<.0001*
CO2-co2 in *CO1* WT	1	76.634	76.634	5.98	0.0196
CO2-co2 in *co1* mut	1	641.479	641.479	50.09	<.0001*

*CO1 and CO2 are highly significant only in the presence of the other mutant allele
