# Supplementary material for: Epistatic interactions between PHOTOPERIOD1, CONSTANS1 and CONSTANS2 modulate the photoperiodic response in wheat
Source: PLoS Genet. 2020 Jul 13;16(7):e1008812. doi: 10.1371/journal.pgen.1008812 (PMC7394450; doi:10.1371/journal.pgen.1008812)
Supplement: S5 Table — (PDF) [file pgen.1008812.s010.pdf]

S5 Table. Primers used in the qRT-PCR and Y2H experiments.

Primer name	Primer Sequence	Genome	Efficiency	Reference
**qRT-PCR**				
SYBR-ACTIN-F	ACCTTCAGTTGCCCAGCAAT		98 %	[1]
SYBR-ACTIN-R	CAGAGTCGAGCACAATACCAGTTG			
TaFT1_AB_qPCR_F	CAGCAGCCCAGGGTTGAG	A & B	97 %	[2]
TaFT1_AB_qPCR_R	ATCTGGGTCTACCATCACGAGTG			
Vrn1-Ex5-6-F2	AAGAAGGAGAGGTCACTGCAGG	A & B	>96 %	[2]
Vrn1-Ex8-R37	GGCTGCACTGCCGCA			
Vrn2_ZCCT2_F	CCACCATCGTGCCATTCT	A & B	>96 %	[1]
Vrn2_ZCCT2_R	CCCACCATCATCTCTGTATCAA			
PPD1_SYB-F3	CGGCATTCACGAGGTACAATAC	A & B	97.6%	[3]
PPD1_SYB-R3	GAGCCTTGCTTCATCTGAGCG			
AC-CO1-AB-F3	CACATCAGAGTGGTTATGC	A & B	94.0 %	[3]
AC-CO1-AB-R3	GGACTGGACCGTATTGTC			
AC-CO2-AB-SYB-F4	AAGGGTGTGAGTGTGTAG	A & B	96.0 %	[3]
AC-CO2-AB-SYB-R5	GATATGTCATTGCTGATGGAAG			
**Y2H** [1]				
PPD1-F [2]	CATATGGACCGTCATCACCAGCAG			
PPD1-R	GAATTCTCTCTCCACGGCAGCCGGCGG			
N-TmPHYB-Y2H-F [3]	**CCGAATTC**ATGGCCTCGGGAAGCCGCGC			
N-TmPHYB-Y2H-F	**TCCCCCCGGG**TGCATCTCTGAAGGAGTCCCG			
C-TmPHYB-Y2H-F	CCGAATTCGGAGAGGGCACTAGTAACTC			
C-TmPHYB-Y2H-F	CCATCGATGCTCCGATCCCTACTTTCTG			

[1] Y2H primers for cloning *PHYC* full-length and truncations and *PHYB* full-length have been described in [3] and those for *CO1*, *CO2* and *VRN2* have been described in [4].

[2] *PPD1* full-length coding region was cloned from Kronos.

[3] *PHYB* clones are from *T. monococcum.* Underlined bases indicate restriction sites used in cloning.

References Table 5

1. Distelfeld A, Tranquilli G, Li C, Yan L, Dubcovsky J. Genetic and molecular characterization of the *VRN2* loci in tetraploid wheat. Plant Physiol. 2009;149(1):245-57. doi: 10.1104/pp.108.129353. PMID: 19005084.
2. Yan L, Fu D, Li C, Blechl A, Tranquilli G, Bonafede M, et al. The wheat and barley vernalization gene *VRN3* is an orthologue of *FT*. Proc Natl Acad Sci U S A. 2006;103(51):19581-6. doi: 10.1073/pnas.0607142103. PMID: 17158798.
3. Chen A, Li C, Hu W, Lau MY, Lin H, Rockwell NC, et al. Phytochrome C plays a major role in the acceleration of wheat flowering under long-day photoperiod. Proc Natl Acad Sci U S A. 2014;111(28):10037-44. doi: 10.1073/pnas.1409795111. PMID: 24961368.
4. Li C, Distelfeld A, Comis A, Dubcovsky J. Wheat flowering repressor VRN2 and promoter CO2 compete for interactions with NUCLEAR FACTOR-Y complexes. Plant J. 2011;67(5):763-73. doi: 10.1111/j.1365-313X.2011.04630.x. PMID: 21554456.
