# Supplementary material for: Epistatic interactions between PHOTOPERIOD1, CONSTANS1 and CONSTANS2 modulate the photoperiodic response in wheat
Source: PLoS Genet. 2020 Jul 13;16(7):e1008812. doi: 10.1371/journal.pgen.1008812 (PMC7394450; doi:10.1371/journal.pgen.1008812)
Supplement: Attachment — Submitted filename: Answers to reviewers and editors questions v3.pdf [file pgen.1008812.s011.pdf]

Answers to Editor and Reviewer's questions (this review is uploaded as an attachment)**.**

Editor requests

1. *I was confused by the nomenclature. The mention of the Ppd1-A1a and Ppd1-A1b backgrounds, then later a mention of ppd1 null. In reading the text, I gather that PpdA1a is a promoter deletion of PPD1 and PpdA1b is ancestral, but it left me puzzled. Perhaps you can guide your naive readers through this in the abstract.*

Author response: We clarified the definition of the two *Ppd-A1* alleles in the abstract: "…acceleration of heading time both in *PHOTOPERIOD1* (*PPD1*) sensitive (*Ppd-A1b*, functional ancestral allele) and insensitive (*Ppd-A1a*, functional dominant allele) backgrounds."

2. *Also you mention Co-1 and CO1 as the wild-type alleles in the abstract (line 36 and 37). That makes it confusing too.*

Author response: we clarified that *Co1* is the functional allele and the *CO1* is the name of the gene, and that *ppd1* indicates loss-of-function mutations in both *PPD1* homeologs in the following modified sentence: "By contrast, in *ppd1* mutants with loss-of-function mutations in both *PPD1* homeologs, the wild type *Co1* allele accelerated heading time >60 d relative to the *co1* mutant under LD."

"We detected significant genetic interactions among *CO1*, *CO2* and *PPD1* genes on heading time…"

3. *I appreciate seeing the inflorescences in figure 3, but the detail of the floral organs is lost without some indication of what we are looking at. Is it simply that the stamens are larger in the WT-PI? Also the use of Waddington's scale is useless to anyone outside of the wheat community I imagine.*

Author response: We labelled the floral organ primordia in the picture and described the differences in detail in the legend. We also added a brief description of the Waddington scale in the legend of Figure 3.

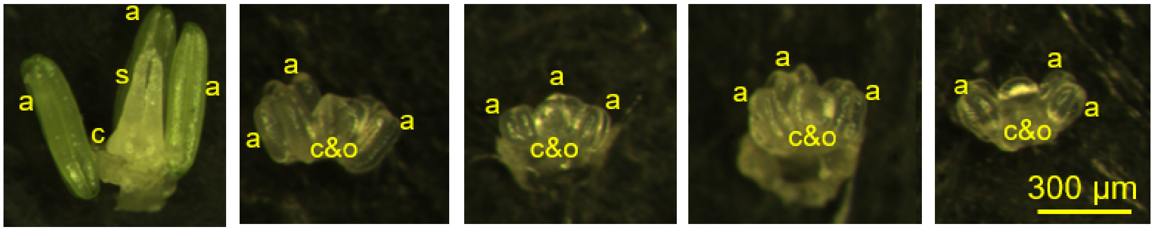

"... a = anthers, elongated in Kronos-PI and primordia in other genotypes, c = carpel and s = styles well developed in Kronos-PI and c&o = carpel extending around the ovule in other genotypes (**B**) Developmental time course of SAM using the Waddington scale from 4- through 11-week-old plants (n = 3). W1 = vegetative apex, W2 = double ridge, W3= glume primordium, W4 = stamen primordium, W5 = carpel extending round three sides of ovule, W6 = short style primordia and stylar canal open, W7 = stigmatic branches elongated, W8 = stigmatic branches elongating, W9 = styles and stigmatic branches erect and stigmatic hairs differentiating."

4. *In Figure 4 B, the col;ppd1 double. What is in the photo? It looks like a spike. As you write, it did not emerge and failed to make grains, but I would say this genotype did flower. Perhaps heading time refers to the emergence of the panicle?*

Author response: Correct, we want to emphasize that plants of all genotypes tested were able to transition to the reproductive stage, but that in LD floral development and spike emergence were arrested in *ppd1 co1*. We clarified that in the legend. We added in Figure 4 B: "Floral transition and spike formation occurred in all genotypes but in the *ppd1 co1* double mutant floral development was delayed or arrested and spikes failed to emerge before the experiment was terminated (heading time > 180 d)."

In Material and Methods we clarified that: "We recorded heading time as the total time from planting in soil to full emergence of the spike from the sheath."

Reviewer #1:

The manuscript by Shaw et al. studies loss of function mutations of PPD1, CO1 and CO2, among others, and their effect on flowering time in wheat. The authors nicely reveal complex genetic and protein-protein interactions among those and other components of the vernalization and photoperiod response pathways. The results are solid and well organized. The Discussion introduces new hypothesis about the genetic control of flowering time in winter cereals. I would like to suggest some minor points that can be revised in the manuscript.
Abstract, line 36 – Specify 'wild-type Co1' accelerated heading time …

Author response: Modified as requested above in the answer to the editor suggestion "By contrast, in *ppd1* mutants with loss-of-function mutations in both *PPD1* homeologs, the wild type *Co1* allele accelerated heading time >60 d relative to *co1* mutant allele under LD."

Figure 1 is a schematic representation of the CO1 gene structure. The gene has two exons, but the figure illustrates a single model, indicating the position of the identified mutations. I guess that stop codon mutations co-A1 and co-B2 are both in exon 1 (line 161), whereas the splice-site mutations co-A2 and co-B1 correspond to the intron, causing intro retention.

Author response: We added two double arrows to the gene model to indicate the location of Exon1 and Exon 2. We also expanded the legend to make more explicit the location of the mutations: "The selected *co-A1* and *co-B2* mutations, located in the first exon after the two B-Box domains, are predicted to generate premature stop codons, whereas the *co-B1* and *co-A2* mutants have acceptor splice-site mutations that result in intron retention and premature stop codons that eliminate the complete exon 2 including the CCT domain"

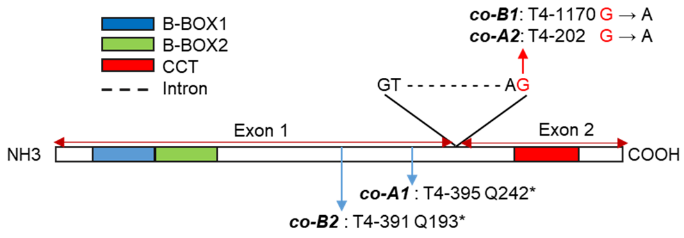

Line 200 – 'The interaction was' instead of 'the interactions was'?

Author response: The typo was corrected to "…the interaction was…"

Lines 761-762 - I understand that the experiment was carried out in CONVIRON growth chambers as the one described in lines 774-776. Unify both sentences within the phenotyping section.

Author response: We combined the description of the chambers in the Phenotyping section: "We evaluated the mutant and control lines in CONVIRON growth chambers with metal halide bulbs supplemented by florescent bulbs set to 22 °C during daylight and 17 °C during dark periods. Lights were set to 260-300 µmol m^{-2} s^{-1} and were on for 16 h in LD experiments and 8 h in short day (SD) experiments". In the expression section we eliminated the description and referred to the conditions described in the previous phenotypic section.

Reviewer #2 (Frank G. Harmon)

This manuscript investigates the role of CONSTANS1 (CO1) and CO2 in photoperiodic control of flowering in tetraploid wheat. co1 and co2 mutant alleles are combined with several different mutant alleles of PHOTOPERIOD1 (PPD1) to understand how these genes interact under long day (LD) and short day (SD) photoperiods. The authors show weak repressive roles for CO1 and CO2, with CO1 being the more important of the two. PPD1 has a comparatively greater role in activating flowering with or without CO1 and CO2. Importantly, CO1 promotes flowering under long days (LD) without functional PPD1, while PPD1 promotes flowering without CO1 or CO2. Gene expression analysis shows complex changes in expression of key flowering time genes that are essentially in line with observed flowering phenotypes. Complex interactions are also demonstrated upon testing of the protein-protein interactions made between CO, PPD1, VRN2, and phytochromes B and C. Because the authors how CO1/2 and PPD1 are functionally independent, this work raises interesting questions about upstream photoperiod perception pathways, as well as how these independent pathways interact downstream. This work also clearly highlights the crucial contribution genetic background makes to the activity of either CO or PPD1, which could be determined by combined transcriptional regulation and a large network of protein-protein interactions.

The work is well done, carefully interpreted, and logically presented with clearly written text.

While the Discussion is primarily focused on events occurring in the leaf to promote FT1 expression (summarized by the working model in lines 640-645), how do these genetic/protein interactions contribute to promotion of spike development in the post-floral transition SAM? This seems like an important consideration, given the authors present in the Introduction that "...in wheat, the photoperiod pathway has a larger impact on the duration of spike development and stem elongation than on the initial transition between the vegetative and reproductive stages" (lines 121-123). Furthermore, the authors show that all Kronos-PI & Kronos-PS genotypes [including co1-PS, co2-PS, and co1 co2-PS] achieved the same post floral transition stage by 6 weeks under SD (Figure 3) before development of Kronos-PI diverged from the others. They also conclude that "PPD1 is not essential for the initial transition of the SAM to the reproductive stage, but in its absence, spike development and stem elongation are severely compromised" (lines 297-299). In total, these observations indicate PPD1 and CO1/2 activity have important contributions to spike development, which presumably occurs following the action of FT1, but how is not explored in the Discussion.

Author response: This was explored in our previous publication Pearce et al. 2013 *Plant Physiology* 163, 1433-1445 [18] and briefly discussed in the introduction:

Under LD conditions, the wheat *PPD1* gene induces the expression of *FT1*, which encodes a mobile protein that travels to the shoot apical meristem (SAM), where it promotes the expression of the floral homeotic MADS box transcription factor *VERNALIZATION1* (*VRN1*) and the synthesis of gibberellic acid (GA) [18]. The simultaneous presence of GA and *VRN1* results in the up-regulation of *SUPPRESSOR OF OVEREXPRESSION OF CONSTANS1-1* (*SOC1*) and *LEAFY* (*LFY*) genes, which are required for normal and timely spike development, stem elongation and spike emergence or heading [18]. In photoperiod sensitive wheats grown under SD, the SAM transitions to the reproductive stage, but stem elongation and spike development proceed very slowly and heads fail to emerge or emerge extremely late [19]. These results indicate that in wheat, the photoperiod pathway has a larger impact on the duration of spike

development and stem elongation than on the initial transition between the vegetative and reproductive stages [18, 19].

We added the following paragraph at the end of the first section of the Discussion: "Although the effect of *CO1* on heading time is limited in the presence of functional *PPD1* alleles, in the *ppd1* mutant background the presence of *CO1* was required for heading under LD (Fig 4). It has been previously shown that hexaploid wheat plants with no functional *PPD1* genes (but with wild type *CO1* alleles) were able to head under LD, and that these plants express *FT1* although at lower levels [24]. These levels should be sufficient to send enough florigen to the SAM to up-regulate both *VRN1* and GA biosynthesis and the subsequent induction of *LFY* and *SOC1*, which are critical for timely spike development and stem elongation [18]. We hypothesize that the inability of the spikes to emerge in the *ppd1 co1* mutants grown under LD or in the Kronos-PS or *ppd1* mutants grown under SD, is associated with insufficient production of *FT1* in the leaves, which results in limited levels of GA, *LFY* and *SOC1* in the SAM. Addition of exogenous GA to photoperiod sensitive *T. monococcum* genotypes that express *VRN1* under SD (due to a deletion in the *VRN1* promoter) has previously been shown to partially rescue the arrest in spike development and stem elongation under SD [18].

For readers outside the temperate grass flowering field, there is too little introduction to the roles of VRN1 and VRN2 to follow the arguments made in the Discussion. The authors could add a few sentences in the Introduction or Discussion to clarify.

Author response: We included in the introduction the following paragraph: "The photoperiod and vernalization pathways converge at the regulation of *FT1* in the leaves. The role of *PPD1* as a LD flowering promoter is antagonized in winter wheats by the role of *VRN2* as a LD flowering repressor [20, 21], which prevents the induction of *FT1* during the fall. Long exposures to cold temperatures during the winter (vernalization) result in the induction of *VRN1* both in the leaves and in the SAM [22]. In the leaves, the presence of *VRN1* prevents the upregulation of *VRN2* in the spring [23], which results in the upregulation of *FT1* under LD. In the SAM, the upregulation of *VRN1* initiates the transition of the SAM to the reproductive phase, but spike development proceeds slowly until the LD induction of *FT1*. The arrival of *FT1* to the SAM further up-regulates *VRN1* and induces GA accumulation, which results in accelerated spike development and stem elongation [18]."

It is not clear what is meant by the inclusion of ELF3 in this context: "..photoperiod upstream of PPD1 and CO1 (e.g. ELF3).." (line 723). It the proposal that ELF3 is the common upstream component of photoperiod perception the regulates PPD1 and CO1/2?

Author response: The BioRxiv manuscript from Gao et al. bioRxiv 10.1101/697169 (2019) suggests that the dark or thermal reversion of the *Pfr* form of PHYC (and maybe PHYB) may be the mechanism underlying the perception of night length in Brachypodium. They show that the ELF3 protein is degraded in the presence of light, and that the protein abundance inversely correlates with the levels of *Pfr.* Since ELF3 functions as a *PPD1* repressor, this study proposes that the ability of PPD1 to perceive day length is mediated by ELF3.

However, we currently do not know if the ability of *CO1* to detect and respond to LD in the absence of PPD1 is mediated or not by ELF3. We know that the *se5* rice mutant that abolishes

the synthesis of chromophore (likely results in non-functional phytochromes) results in early flowering in rice. The rice *se5* and *hd1* single mutants flower as early as the *hd1 se5* double mutant suggesting that these genes function in the same pathway, but we currently do not know if CO1 can perceive photoperiod independently of ELF3. We are currently generating the double mutants required to test this hypothesis, but it will take some time, so we cannot include it in this story.

We favor the hypothesis that temperate grasses have two independent pathways to measure photoperiod: one including *PHYC-PHYB / ELF3 / PPD1* that follows the hourglass model (with the dark reversion of Pfr as the sand measuring the time, as described by Gao et al. 2019) and the other one including CO1/CO2 that follows the external coincidence model as described for CO in Arabidopsis. However, we cannot rule out the possibility that, in the temperate grasses, CO1 ability to measure photoperiod is also mediated by ELF3.

We added the following paragraph to describe better the results from Gao et al 2019 and clarify the potential role of ELF3. "Recently, it has been suggested that the dark reversion of the Pfr form of PHYC may be involved in measuring night length (molecular hourglass model), by modulating the accumulation of the ELF3 protein, a direct transcriptional repressor of *PPD1* [35]. This mechanism is different from the external coincidence model proposed for Arabidopsis [66]. Although we favour the hypothesis of separate mechanisms for *PPD1* and *CO1* perception of photoperiod, we currently cannot rule out the possibility that, in the temperate grasses, ELF3 mediates both the *PPD1* and *CO1* responses to photoperiod."
